# TRH-induced secretion of adrenocorticotropin and cortisol in dogs with pituitary-dependent hypercortisolism

**DOI:** 10.1080/01652176.2018.1521537

**Published:** 2018-10-26

**Authors:** Tera Pijnacker, Marieke Knies, Sara Galac, Karin Sanders, Jan A Mol, Hans S Kooistra

**Affiliations:** Department of Clinical Sciences of Companion Animals, Faculty of Veterinary Medicine, Utrecht University, Utrecht, The Netherlands

**Keywords:** Canine, dog, Cushing, diagnosis, TRH receptor, immunohistochemistry

## Abstract

**Background:** In dogs, spontaneous Cushing’s syndrome is most often pituitary-dependent and caused by hypersecretion of adrenocorticotropic hormone (ACTH), resulting in increased adrenocortical glucocorticoid secretion similar to horses. In horses with Cushing’s syndrome (or pituitary pars intermedia dysfunction [PPID]) a thyrotropin-releasing hormone (TRH) stimulation test can be used for diagnosis, as TRH administration results in increased circulating ACTH and cortisol concentrations in affected horses.

**Objective:** The aim of this study was to investigate the effect of TRH administration on the circulating ACTH and cortisol concentrations in dogs with pituitary-dependent hypercortisolism (PDH).

**Methods:** Ten clinically normal control dogs and 10 dogs with PDH, all client owned, underwent a TRH stimulation test with measurement of plasma concentrations of ACTH and cortisol, before and after intravenous administration of 10 μg TRH/kg bodyweight.

**Results:** Plasma ACTH concentration did not rise significantly after TRH stimulation, neither in PDH dogs nor in clinically normal dogs. In contrast, the plasma cortisol concentration did increase significantly after TRH stimulation in both groups (*p* = .003 in PDH and *p* < .001 in control). Immunohistochemistry of normal adrenal glands demonstrated the presence of TRH receptors in the whole adrenal cortex.

**Conclusions:** The results of this study demonstrate that the TRH stimulation test should be rejected as a tool to diagnose PDH in dogs. The observed TRH-induced increase in plasma cortisol concentration without a significant rise in plasma ACTH concentration may be explained by a direct effect of TRH on adrenocortical cells mediated by adrenocortical TRH receptors.

## 1. Introduction

Spontaneous hypercortisolism or Cushing’s syndrome is one of the most common endocrinopathies in dogs, with a reported incidence of 1–2 per 1000 dogs per year (Willeberg and Priester 1982). Clinical signs reflect the effects of glucocorticoid excess, and comprise centripetal obesity, muscle and skin atrophy, polyuria and polyphagia. In 15–20% of the dog’s hypercortisolism is caused by an adrenocortical adenoma or carcinoma, autonomously secreting glucocorticoids. In the other 80–85% of cases, canine hypercortisolism is pituitary-dependent and caused by hypersecretion of adrenocorticotropic hormone (ACTH), resulting in increased glucocorticoid secretion by the adrenals (Owens and Drucker 1977). Approximately 75–80% of dogs with pituitary-dependent hypercortisolism (PDH) have an ACTH producing adenoma located in the anterior lobe (AL) of the pituitary gland; in the other 20–25% the adenoma arises from the pars intermedia (PI) (Peterson et al. 1982; Peterson et al. 1986). Currently, the endocrine diagnosis of canine hypercortisolism is commonly based on the results of a low-dose dexamethasone suppression test (LDDST) or an ACTH stimulation test, or on measurement of the urinary corticoid-to-creatinine ratio (UCCR) (Kooistra and Galac 2012; Behrend et al. 2013). However, the ACTH stimulation test has suboptimal sensitivity and specificity, and synthetic ACTH is not easily available in every country. The LDDST has the disadvantage of taking 8 h, whereas urine samples for determination of the UCCR have to be collected by the owner and the UCCR can be false-positive due to stress and non-adrenal disease.

In horses, Cushing’s syndrome is also a very common endocrinopathy. In this species, it is also known as pituitary pars intermedia dysfunction (PPID), because the disorder almost exclusively originates from the PI (Schott 2002; McFarlane et al. 2006). A thyrotropin-releasing hormone (TRH) stimulation test can be used to diagnose PPID in equines. Although TRH administration results in increased circulating concentrations of ACTH and cortisol in both healthy horses and horses with Cushing’s syndrome, animals with PPID respond with a larger increment of ACTH and cortisol (Thompson et al. 1995; McFarlane et al. 2006). McFarlane et al. (2006) demonstrated the expression of TRH receptor (TRHR) mRNA in the PI in both normal and PPID horses. They concluded that the TRH-induced increase in cortisol secretion was secondary to TRHR-mediated ACTH secretion of the PI. A similar response has been reported in humans with pituitary-dependent Cushing’s syndrome, where some patients showed an increased response in ACTH and cortisol after TRH administration (Krieger and Luria 1977; Pieters et al. 1979). Moreover, human pituitary adenoma tissue in culture also responded to TRH with release of ACTH, whereas normal pituitary tissue did not (Ishibashi and Yamaji 1981). These findings suggest aberrant TRHR expression in human pituitary adenomatous ACTH-producing cells.

This study investigated the effect of TRH administration on circulating concentrations of ACTH and cortisol, in both clinically normal control dogs and dogs with PDH, in order to evaluate whether a TRH stimulation test may be used to diagnose hypercortisolism in dogs.

## 2. Materials and methods

### 2.1. Dogs

Ten client-owned dogs with PDH of nine different breeds (Golden retriever, Mongrel, Bull Mastiff, Toy Poodle, Dachshund, Basset, Beagle (2), Labrador retriever, Airedale terrier) were included in the study. The dogs’ ages ranged from 7 to 14 years, with a median age of 11 years. Median body weight was 18.8 kg, with a range of 9-57 kg. Two dogs were male (all neutered) and eight female (all spayed). All dogs had a history, physical changes, and biochemical and hematological findings consistent with hypercortisolism. The endocrine diagnosis of hypercortisolism was based on elevated UCCRs in morning urine samples collected on two consecutive days at home, at least 2 d after the last visit to the veterinary practice (median 26 × 10^−6^, range 15 to 203 × 10^−6^, reference <10 × 10^−6^) (Kooistra and Galac 2012; Behrend et al. 2013). After collection of the second urine sample, three doses of 0.1 mg/kg bodyweight dexamethasone were administered orally at intervals of 6–8 h (Kooistra and Galac 2012). The UCCR in the third urine sample of all dogs was less than 50% of the mean of the first two samples, consistent with PDH (Galac et al. 1997). None of the dogs were treated for their hypercortisolism at the time of the TRH stimulation test.

Ten client-owned clinically normal dogs of nine different breeds (Fox terrier, Staffordshire bullterrier, Golden retriever (2), Belgian shepherd dog, American Stafford, Jack Russel terrier, Dutch shepherd dog, Bernese Mountain dog, and mongrel) were included in the study as controls. The dogs were healthy dogs either presented for vaccination or pets of the staff. The dogs’ ages ranged from 6 to 13 years, with a median age of 7.5 years. Median body weight was 26 kg, with a range of 7–40 kg. Three dogs were male (all neutered) and seven female (all spayed). The control dogs did not have clinical signs of hypercortisolism and all had UCCRs on two consecutive days within the reference range (all values between 0.2 × 10^−6^ and 8.2 × 10^−6^) and a urine specific gravity of >1.025.

The study was approved by the Ethical Committee of Utrecht University and all owners signed an informed consent.

### 2.2. TRH stimulation test

Blood samples were collected at −15, 0, 10, 20, and 90 min after intravenous administration of 10 μg/kg of TRH^a^ (TRH, uncompounded, Ferring Arzneimittel GmbH, Protirelin 0.2 mg, Kiel, Germany). The samples were collected by jugular venipuncture and immediately transferred into chilled heparin-coated tubes for analysis of the plasma concentrations of cortisol, thyroxine (T_4_) and TSH, and chilled EDTA-coated tubes for plasma ACTH concentration analysis. Within 15 min after collection, the blood samples were centrifuged for 5 min at 3000×*g* and plasma was stored at −20 °C until analyzed.

Plasma cortisol and ACTH concentrations were measured at −15, 0, 10, 20, and 90 min after TRH stimulation, plasma T_4_ concentration was measured at −15 and 90 min post TRH stimulation, and plasma TSH concentration at −15, 0, and 20 min in the PDH dogs and at −15, 0, and 90 min in the control dogs.

The areas under the curve (AUC) for plasma cortisol and ACTH were calculated using the trapezoidal rule (Jordan and Smith 2008).

### 2.3. Assays

The urinary corticoid concentration was measured by radioimmunoassay (RIA) as described previously (Galac et al. 2009). The intra- and inter-assay coefficients of variation were 6 and 8%, respectively. The sensitivity was 1 nmol/L. The urinary corticoid concentration was related to the urinary creatinine concentration (Jaffé kinetic method, initial rate reaction) and the UCCR was calculated.

Plasma cortisol concentration was determined with a homologous solid-phase, chemiluminescence enzyme immunoassay (Immulite 2000; Siemens Healthcare Diagnostics, Den Haag, The Netherlands). The intra- and inter-assay coefficients of variation were 7.4 and 9.4%, respectively. The sensitivity was 5.5 nmol/L.

Plasma ACTH concentration was measured using a solid-phase, two-site sequential chemiluminescent immunoradiometric assay (Immulite 2000; Siemens Healthcare Diagnostics, Den Haag, The Netherlands). The antiserum is highly specific for ACTH (1–39). The intra- and inter-assay coefficients of variation were 3.2 and 7.8%, respectively. The sensitivity was 0.22 pmol/L.

Plasma T_4_ concentration was determined with a homologous solid-phase, chemiluminescence enzyme immunoassay (Immulite 2000 Total T_4_^®^; Siemens Healthcare Diagnostics, Den Haag, The Netherlands) in accordance with the instructions of the manufacturer. The intra-assay coefficients of variation were 13.8% and 8.2% at plasma T_4_ concentrations of 8 and 25 nmol/L, respectively. The inter-assay coefficient of variation was 8.5% at a plasma T_4_ concentration of 21 nmol/L. The lowest detectable concentration of T_4_ was 2 nmol/L.

Plasma TSH concentration was determined by a homologous solid-phase, two-site chemiluminescent enzyme immunometric assay (Immulite 2000 canine TSH^®^, Siemens Healthcare Diagnostics, Den Haag, The Netherlands), in accordance with the instruction of the manufacturer and as described previously (Bruner et al. 1998). The intra-assay coefficients of variation were 5.0 and 4.0% at TSH concentrations of 0.20 and 0.50 µg/L, respectively. The inter-assay coefficient of variation was 6.3% at a TSH concentration of 0.16 µg/L. The lowest detectable concentration of TSH was 0.03 µg/L.

### 2.4. Adrenal and pituitary gland tissues

Tissues were available as archived tissue and their use was approved by the Ethical Committee of Utrecht University. For immunohistochemistry, the adrenal glands of eight clinically healthy dogs were used. After resection, the tissues were fixed in 4% buffered formaldehyde for 24–48 h, embedded in paraffin, cut into 5 µm sections and mounted on SuperFrost Plus microscope slides (Menzel-Gläser, Braunschweig, Germany). Histopathologically, all adrenals were judged as normal. For the Western blot analysis, one adrenal cortex and the complete pituitary gland of one clinically healthy dog were used, which were snap frozen in liquid nitrogen within 10 min after resection and kept at −70 °C until further use.

### 2.5. Western blot

A Western blot was performed to confirm the specificity of the anti-TRHR antibody. Protein was isolated from a normal canine pituitary and a normal canine adrenal gland using radioimmunoprecipitation buffer base. Total protein concentrations were measured using the DC™ Protein Assay (BioRad, Veenendaal, the Netherlands), and the protein homogenates were subsequently diluted with purified water to 2 µg/µL. The samples were diluted 1:1 with sample buffer and heated at 95 °C for 2 min. Then, 20 µL of the diluted samples (1 μg protein/μL) or 5 µL of the Precision Plus Protein Standard (BioRad, Veenendaal, the Netherlands) was loaded onto a 4–20% Criterion™ TGX™ Precast Midi Protein Gel (BioRad, Veenendaal, the Netherlands) and gel-electrophoresis was performed.

Afterward, the gel was blotted onto a Hybond enhanced chemiluminescence (ECL) nitrocellulose membrane (Amersham, GE Healthcare, Diegem, Belgium). The membrane was blocked for 60 min in Tris-buffered saline with 0.1% Tween (TBST 0.1%) with 4% ECL Blocking Agent (Amersham, GE Healthcare, Diegem, Belgium), and incubated overnight at 4 °C with the anti-TRHR antibody (rabbit polyclonal, ab72179, Abcam, Cambridge, UK) in a 1:500 concentration (1 µg/mL), diluted in 4% bovine serum albumin (BSA) in TBST 0.1%.

The following day, the membrane was incubated for 60 min with a secondary antibody (anti-rabbit, horseradish peroxidase conjugated, 1:20,000). All washing steps were performed with TBST 0.1%. An ECL advanced Western blotting detection kit (Amersham, GE Healthcare, Diegem, Belgium) was used for protein visualization and chemiluminescence was detected using a ChemiDoc XRS Chemi Luminescent Image Capture (BioRad, Veenendaal, the Netherlands).

After visualization, the membrane was stripped with Restore™ Western Blot Stripping Buffer (Thermo Scientific, Naarden, the Netherlands) to stain for Actin as a loading control. The membrane was subsequently again blocked with ECL Blocking Agent as described above, and incubated overnight at 4 °C with an anti-Actin, pan Ab-5 antibody (MS-1295-P1, Thermo Scientific, Naarden, the Netherlands) in a 1:2000 concentration (0.1 µg/mL), diluted in 4% BSA in TBST 0.1%. The following day, the membrane was incubated for 60 min with a secondary antibody (anti-mouse, horseradish peroxidase conjugated, 1:20,000). All washing steps were performed with TBST 0.1%. An ECL advanced Western blotting detection kit (Amersham, GE Healthcare, Diegem, Belgium) was used for protein visualization, and chemiluminescence was detected using a ChemiDoc XRS Chemi Luminescent Image Capture (BioRad, Veenendaal, the Netherlands).

### 2.6. Immunohistochemistry for detection of TRHR

Immunohistochemical staining was performed on formalin-fixed, paraffin-embedded tissue slides. Tissue slides were rehydrated in a series of xylene and alcohol baths. For antigen retrieval, the slides were kept at 98 °C for 10 min in a Tris-EDTA buffer. The slides were blocked for 30 min with 10% normal goat serum, diluted in TBS with 1% BSA and 0.025% Triton X-100. The slides were incubated overnight at 4 °C with TRHR antibody (ab72179, Abcam, Cambridge, UK) at a dilution of 1:100 (5 µg/mL) in TBS with 1% BSA and 0.025% Triton X-100.

The following day, the slides were immersed in 0.35% H_2_O_2_ peroxidase for 10 min to block endogenous peroxidases. A secondary goat anti-rabbit antibody (Envision, K4003, DAKO, Heverlee, Belgium) was applied for 30 min. All washing steps were performed with TBS with 0.025% Triton X-100. Bound antibody was visualized with the DAB substrate kit for peroxidase (K3468, Dako, Heverlee, Belgium), after which the slides were counterstained with hematoxylin, dehydrated in a series of ethanol and xylene baths, and embedded in a mounting medium.

## 3. Statistical analysis

For the statistical analysis, a commercially available standard statistic software (IBM SPSS Statistics version 24, IBM corp., NY) was used. Normal distribution of data was assessed using the Kolmogorov–Smirnov test. Gender and neutering status were tested with a chi-squared test. Body weight and age were compared with an independent samples *t*-test. Differences between basal plasma levels (average of t= −15 and *t* = 0) and levels at *t* = 20, *t* = 30, *t* = 45 or *t* = 90 min were tested with a Friedman’s ANOVA test. When significant a Wilcoxon signed-rank test (with Bonferroni correction for multiple comparison) was used as *post-hoc* test. Differences in hormone values (including the AUC) between PDH dogs and control dogs were compared with a Mann–Whitney U test. Differences were considered significant at *p* < α/m with α = 0.05 and m set by the appropriate Bonferroni correction. Data are presented as median and range. All Western blot results and immunohistochemistry slides were analyzed in a descriptive manner.

## 4. Results

There were no significant differences in baseline characteristics (gender, neutering status, bodyweight or age) between the PDH dogs and control dogs. TRH administration did not reveal any side effects.

**Figure 1.**
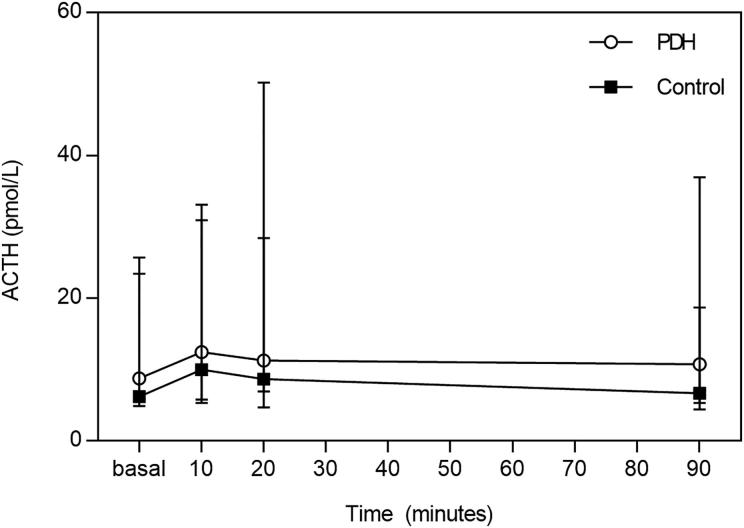
Plasma ACTH concentrations (pmol/L), median and range, at baseline and 10, 20, and 90 min after TRH administration in 10 dogs with PDH (open circles) and 10 control dogs (squares).

The median basal plasma ACTH concentration in the PDH dogs (8.8 pmol/L; range, 6.3–25.7 pmol/L) did not differ significantly (*p* = .075) from that in the control group (6.2 pmol/L; range, 4.9–23.4 pmol/L). Also, the plasma ACTH AUC did not differ significantly between the control group and PDH dogs (*p* = .326). The plasma ACTH concentration did not change significantly (*p* = .99) in the PDH group after administration of TRH. Although the plasma ACTH concentration did change significantly (*p* = .035) in the control group after administration of TRH, *post-hoc* analysis revealed no significant change in plasma ACTH concentration between individual time points (Figure 1).

**Figure 2.**
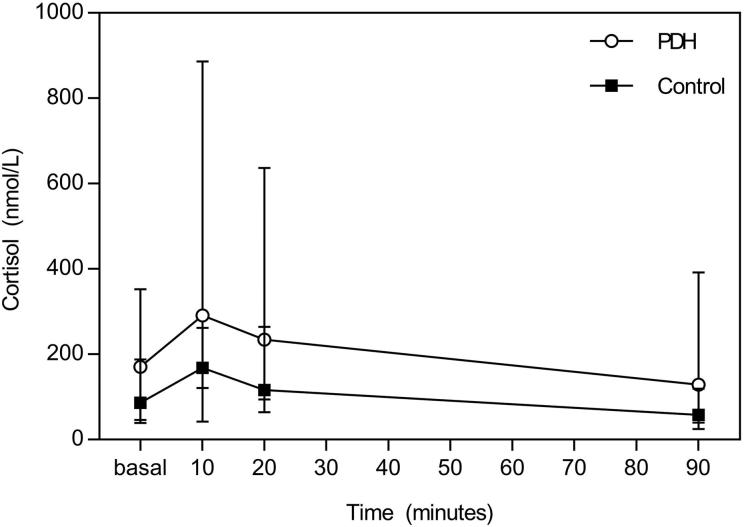
Plasma cortisol concentrations (nmol/L), median and range, at baseline and at 10, 20, and 90 min after TRH administration in 10 dogs with PDH (open circles) and 10 control dogs (squares). The plasma cortisol concentration changed significantly (*p* = .003) in the PDH group after administration of TRH, and *post-hoc* analysis revealed a significant difference between basal and *t* = 10 (*p* = .013), *t* = 10 and *t* = 20 (*p* = .009) and *t* = 20 and *t* = 90 (*p* = .011). The plasma cortisol concentration also changed significantly (*p* < .001) after TRH administration in the control group, and *post-hoc* analysis revealed a significant difference between basal and *t* = 10 (*p* = .007) and between *t* = 20 and *t* = 90 (*p* = .007).

The median basal plasma cortisol concentration in the PDH dogs (170 nmol/L; range, 46–352 nmol/L) did not differ significantly (*p* = .063) from that in the control group (86 nmol/L; range, 39–188 nmol/L). Also, the plasma cortisol AUC did not differ significantly between the control group and PDH dogs (*p* = .131). The plasma cortisol concentration changed significantly (*p* = .003) in the PDH group after administration of TRH (Figure 2). *Post-hoc* analysis revealed a significant difference between basal and *t* = 10 (*p* = .013), *t* = 10 and *t* = 20 (*p* = .009), and *t* = 20 and *t* = 90 (*p* = .011), with Bonferroni correction for multiple comparison. The plasma cortisol concentration also changed significantly (*p* < .001) after TRH administration in the control group (Figure 2). *Post-hoc* analysis with Bonferroni correction for multiple comparison revealed a significant difference between basal and *t* = 10 (*p* = .007) and between *t* = 20 and *t* = 90 (*p* = .007).

The median basal plasma TSH concentration in the PDH dogs (0.23 μg/L; range, 0.11–0.38 μg/L) was significantly higher (*p* = .023) than in the control group (0.10 μg/L; range, 0.03–0.41 μg/L). The plasma TSH concentration increased significantly (*p* < .001) in both groups after administration of TRH.

The median basal plasma T_4_ concentration in the PDH dogs (10 nmol/L; range, 4–29 nmol/L) was significantly lower (*p* = .015) than in the control group (19 nmol/L; range, 10–25 nmol/L). The plasma T_4_ concentration increased significantly (*p* < .001) in both groups after administration of TRH.

**Figure 3.**
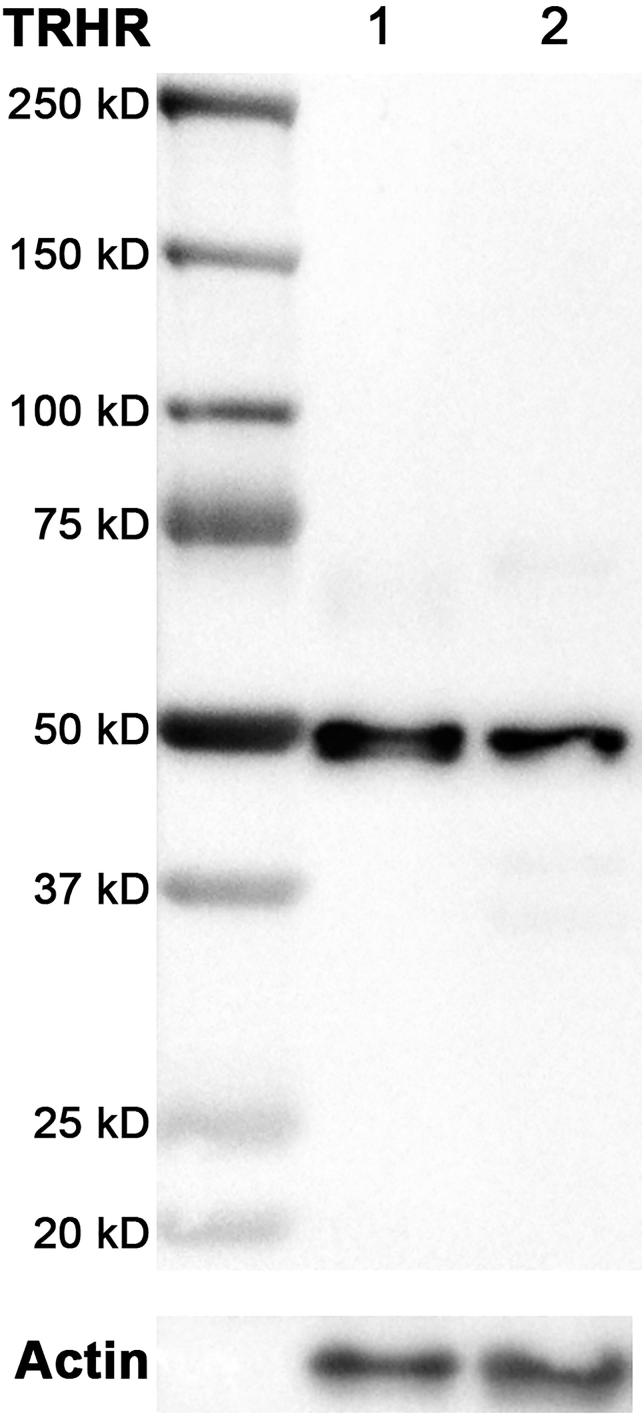
Western blot of TRHR and Actin. Lane 1: normal canine pituitary homogenate; lane 2: normal canine adrenal cortex homogenate. The signal for TRHR was apparent at approximately 50 kDa. Actin was used as a loading control, and its signal was apparent at approximately 45 kDa.

**Figure 4.**
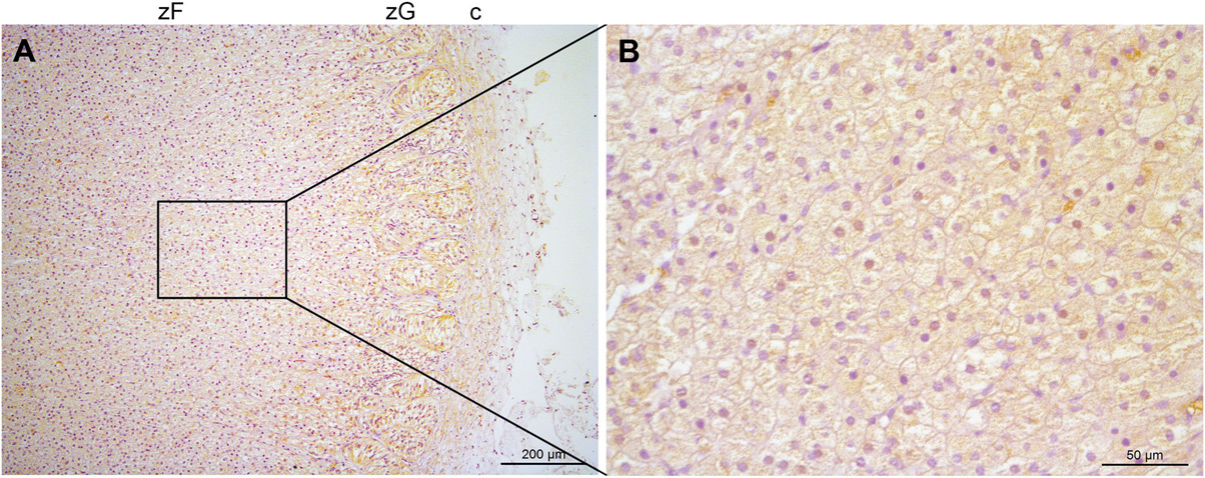
Immunohistochemical staining of thyrotropin-releasing hormone receptor (TRHR). (A) Representative example of immunohistochemical staining for TRHR in a normal adrenal gland. Staining is present in the zona glomerulosa, zona fasciculata and zona reticulata in a membranous and cytoplasmic granular pattern. Bar represents 200 μm. (B) Magnification with 40x objective of the zona fasciculata. Bar represents 50 μm. c: capsule; zG: zona glomerulosa; zF: zona fasciculata.

The Western blot revealed a band at approximately 50 kD, corresponding to TRHR’s expected weight, in both the pituitary and the adrenal tissues, confirming the suitability of the anti-TRHR antibody for use in dogs (Figure 3). Immunohistochemistry of the normal adrenal glands demonstrated the presence of the TRHR in the whole adrenal cortex. A membranous and cytoplasmic pattern was observed in all zones, being most prominent in the zona fasciculata and zona reticularis (Figure 4).

## 5. Discussion

The results of our study show that TRH administration did not result in a significant increase in plasma ACTH concentration, neither in control dogs nor in dogs with PDH. The observed increase in plasma T_4_ and TSH after TRH administration shows a biological effect of TRH in both control dogs and PDH dogs.

The difference in TRH-induced ACTH secretion in horses with PPID and the dogs in this study with PDH may be explained by the fact that none of the PDH dogs included in this study obviously had a PI tumor. All PDH dogs responded with a decrease in UCCR of more than 50% after oral administration of high doses of dexamethasone, indicating that a pituitary AL tumor was the cause of the hypercortisolism. In the pituitary-adrenal cortex, axis cortisol receptors are only expressed in the AL. Consequently, a significant decrease in cortisol secretion in dogs with hypercortisolism can only be found in cases of a corticotroph adenoma in the AL (Galac et al. 1997).

The fact that TRH administration did not result in a significant increase in plasma ACTH concentration in dogs with PDH, indicates that the TRH stimulation test is not a suitable screening test for dogs with PDH. It would be interesting to know whether TRH administration would result in an increase in plasma ACTH concentration in dogs with PDH due to a PI tumor. However, it is difficult to differentiate PI tumors from AL tumors without histopathological examination (including immunohistochemical staining for alpha-melanocyte-stimulating hormone) in dogs.

Interestingly, although TRH administration did not result in a rise in plasma ACTH concentration, TRH administration did result in a significant increase in plasma cortisol concentration, both in control dogs and dogs with PDH. This is in accordance with another study who also reported an increase in circulating cortisol concentrations after TRH administration in healthy dogs and dogs with PDH (Stolp et al. 1982). In contrast, healthy humans do not show a TRH-induced increase in circulating cortisol concentration, whereas in some people with PDH administration of TRH does result in an increased plasma cortisol concentration (Krieger and Luria 1977; Pieters et al. 1979). Originally, it was hypothesized that humans with TRH-induced secretion of cortisol would have a PI tumor, comparable to PPID in horses. However, a later study did not demonstrate that Cushing patients with TRH-induced cortisol secretion were more likely to have a PI tumor (Pieters et al. 1982).

The TRH-induced secretion of cortisol in our dogs was not accompanied by a significant rise in ACTH secretion. It is possible that a TRH-induced increase in ACTH secretion was not detected as significant because of the well-known high variation in ACTH secretion (Kooistra et al. 1997) combined with a possible lack of statistical power in this study.

An alternative explanation for a significant rise in cortisol without a significant rise in ACTH could be aberrant expression of functional TRHRs in the adrenal cortex. We demonstrated for the first time the protein expression of the TRHR in both zones of normal canine adrenocortical tissue. Aberrant expression of several hormone receptors in the adrenal cortex, particularly G-protein coupled receptors, has been reported as a regulating mechanism of cortisol production when ACTH of pituitary origin is suppressed (El Ghorayeb et al. 2015). The best-characterized example to date is the aberrant expression of the gastric-inhibitory polypeptide receptor (GIPR) that may cause food-dependent hypercortisolism (N’Diaye et al. 1998; Galac et al. 2008). Next, to GIP, steroidogenesis can be driven by the expression of ectopic receptors such as serotonin, glucagon, beta-adrenergic receptors, and vasopressin receptors V2 and V3, as well as malfunction of eutopic luteinizing hormone receptors vasopressin receptor V1, or serotonin receptor (El Ghorayeb et al. 2015). In humans, ectopic TRHR expression has been detected in aldosterone-secreting adrenal tumors and aldosterone responsiveness to TRH has been demonstrated in a subset of patients with primary aldosteronism (Zwermann et al. 2009). In dogs, further studies are needed to investigate the link between the TRHR and steroidogenesis in adrenal disorders.

In conclusion, the results of this study demonstrate that the TRH stimulation test should be rejected as tool to diagnose PDH in dogs. The observed TRH-induced increase in plasma cortisol concentration without a significant rise in plasma ACTH concentration may be explained by a direct effect of TRH on adrenocortical cells mediated by adrenocortical TRHRs.

## Disclosure of interest

The authors report no conflict of interest or use of off-label antimicrobials.
